# Autoimmune Hepatitis and Drug-Induced Liver Injury in Japan

**DOI:** 10.3390/jcm14134514

**Published:** 2025-06-25

**Authors:** Hiroki Nishikawa, Soo Ki Kim, Akira Asai

**Affiliations:** 1 Second Department of Internal Medicine, Osaka Medical and Pharmaceutical University, Takatsuki 569-8686, Osaka, Japan; 2 Department of Gastroenterology, Kobe Asahi Hospital, Kobe 653-8501, Hyogo, Japan; kinggold@kobe-asahi-hp.com

**Keywords:** autoimmune hepatitis, drug-induced liver injury, pathogenesis, diagnosis, treatment, Japan

## Abstract

Autoimmune hepatitis (AIH) is the most common liver disease caused by autoimmunity. In Japan, the number of patients with AIH has been increasing in recent years. AIH develops as a result of the loss of immune tolerance to autoantigens in the liver. Drug-induced liver injury (DILI) is an extremely important cause of liver injury in clinical practice and should always be kept in mind in the differential diagnosis. Recently, DILI caused by immune checkpoint inhibitors has been attracting attention. For the diagnosis of DILI, it is important to carefully exclude other possible causes of liver injury and obtain a detailed history of medications and the timing of their use. On the other hand, drug-induced AIH, like hepatitis, also exists and is clinically important because it is often difficult to differentiate from idiopathic AIH. A solid understanding of the pathogenesis of both AIH and DILI is essential for clinicians. This article provides an overview of AIH and DILI in Japan, including the latest findings.

## 1. Introduction

Autoimmune hepatitis (AIH) is the most common liver disease caused by autoimmunity [1,2]. When genetically predisposed individuals are exposed to certain triggering factors, suppression is released, and self-reactive T and B cells proliferate and differentiate into memory cells and effector cells [1,2]. They then accumulate in the liver, leading to the loss of immune tolerance to autoantigens and the development of AIH [1,2]. Drug-induced liver injury (DILI) is an extremely important cause of liver injury in clinical practice and should always be kept in mind in the differential diagnosis [3]. The most common suspect drugs are acetaminophen, antimicrobials, anti-inflammatory drugs, and neuropsychiatric drugs. A number of DILIs due to herbal medicines and health and natural foods have also been reported. Recently, DILI with immune checkpoint inhibitors (ICIs) has attracted attention. DILI is classified into hepatocellular damage type, bile stasis type, and mixed type according to liver enzyme levels [3]. For the diagnosis of DILI, it is important to carefully exclude other possible causes of liver injury and obtain a detailed history of medications and the timing of their use [3]. On the other hand, drug-induced AIH, like hepatitis, (DI-ALH) also exists and is clinically important because it is often difficult to differentiate from idiopathic AIH [4]. In addition, AIH may be complicated by DILI, or AIH may develop following DILI, making it difficult to distinguish between the two in many cases. A solid understanding of the pathogenesis of both is thus essential for clinicians. This article provides an overview of AIH and DILI in our country, along with the latest findings.

## 2. AIH

### 2.1. Epidemiology of AIH: Trends in Japan

Both genetic and environmental factors are thought to play a role in the development of AIH. An important genetic factor is HLA-DR4, a disease susceptibility gene, and about 70% of AIH cases in Japan are HLA-DR4-positive [5]. On the other hand, the positivity rate of HLA-DR3, a disease susceptibility gene for AIH in Europe and the United States, is almost zero in Japan [6]. Viral infection, drugs, and other environmental factors are thought to be involved in the development of AIH, and cases have been reported in which the disease is triggered by infection with EB virus, herpes virus, or hepatitis A virus or following DILI. Therefore, it is important to obtain a detailed history of medications (including health foods) and a medical history. It is also important to note that pregnancy and childbirth may trigger the onset of AIH [5].

In Japan, the number of patients with AIH has been increasing, partly reflecting clearer diagnostic criteria for AIH; a Japanese epidemiological survey in 2016 reported an estimated 30,325 cases, with a prevalence rate of 23.9 per 100,000 population [1]. Compared with the 2004 survey, the number of patients with AIH in Japan increased about threefold, and the male-to-female ratio increased from 1:6.9 to 1:4.3, indicating an increase in male patients [1]. In a nationwide survey of AIH conducted by the Japanese Ministry of Health, Labour, and Welfare research group in 2018 (884 newly diagnosed AIH cases from January 2014 to December 2017), the male-to-female ratio was 1:5.1, and the age at diagnosis was 60.2 years. The absolute number of AIH cases has also been increasing in recent years, and with the current aging of the population in Japan, more AIH cases are occurring at an older age than before [7]. It should be noted, however, that AIH can occur at any age [1]. In addition, AIH is usually chronic active hepatitis, but AIH that develops like acute hepatitis (acute-onset AIH) has become a clinical problem [8,9]. DILI and DI-ALH are sometimes difficult to differentiate [10]. It is important to review the patient’s medical history, including medication history and health foods consumed. Drugs known to induce AIH include minocycline, infliximab, statins, interferon, fenofibrate, etc. [10].

### 2.2. Pathogenesis of AIH

AIH represents a liver disease of autoimmune origin. When genetically predisposed individuals are exposed to certain triggering factors, suppression is released, and self-reactive T and B cells proliferate and differentiate into memory cells and effector cells [1,2,11]. They then accumulate in the liver, eventually leading to a loss of immune tolerance to self-antigens and the development of AIH [1,2,11]. In AIH, abnormal function of toll-like receptors (TLRs) and the activation of dendritic cells by damage-associated molecular patterns (DAMPs) in innate immunity have been shown [2]. AIH has a high prevalence of antibodies that react with hepatocyte membranes, and antibody-dependent cell cytotoxicity is also involved in the pathogenesis of AIH [1,2]. In acquired immunity, quantitative and qualitative abnormalities of regulatory T cells and increases in IL-17 and Th17 cells have been shown [2]. In a study of cytokines and chemokines, serum IL-21 was reported to correlate with the degree of inflammation and serum IgG levels. In a very acute presentation or fulminant AIH, AIH-related characteristic IgG elevation may be missing, indicating that this immunological abnormality is more a consequence of AIH rather than related to the cause of AIH, further supporting the concept that AIH is primarily a T cell-driven disease and is not mediated by autoantibodies [12]. The high rate of relapse in AIH cases with high serum IL-33 levels before treatment has been suggested to predict relapse of AIH [13]. IL-33 is produced by hepatocytes and endothelial cells and is positively correlated with IL-17, IFN-γ, and IL-21 [13]. Serum B-cell activating factor belonging to the tumor necrosis factor family (BAFF) levels and serum interferon-γ-inducible protein-10 (IP-10) levels in AIH can be useful for predicting the degree of liver inflammation activity in AIH [14].

### 2.3. Diagnosis of AIH

#### 2.3.1. AIH, Autoantibodies, and Pathology

AIH is classified into type 1 and type 2 according to the detection pattern of autoantibodies, with type 1 being more common in Japan and positive for antinuclear or anti-smooth muscle antibodies. Smooth muscle antibody is an autoantibody against actin, one of the muscle protein components [5]. Type 2 is positive for anti-liver kidney microsome-1 (LKM-1) antibodies, etc., and it is particularly common in young patients in Europe and the United States but extremely rare in Japan [5]. Most AIH cases in Japan are positive for anti-nuclear antibodies, anti-smooth muscle antibodies, or both. However, both antibodies have low disease specificity, and nuclear antigens specific for AIH have not been identified. If both anti-nuclear and anti-smooth muscle antibodies are negative, anti-LKM-1 antibody should be measured. As mentioned above, HLA-DR4-positive cases are more frequent in Japan. Anti-soluble liver antigen (SLA) antibody is the only AIH-specific autoantibody and is found in 20–30% of both type 1 and type 2 AIH [5]. Anti-neutrophil cytoplasmic antibody is frequently positive in type 1 AIH and is associated with inflammatory bowel disease and with primary/autoimmune sclerosing cholangitis [15]. Anti-SLA antibody-positive cases have been reported to have a better response to treatment compared to negative cases [16]. Non-alcoholic fatty liver disease often requires differentiation from AIH because of positive anti-nuclear antibodies, but most can be easily differentiated by liver biopsy [17]. AIH is characterized by higher serum IgG levels. In most cases, serum IgG is greater than 2.0 g/dL, but a recent nationwide survey in Japan found that many cases (38.9%) were under 2.0 g/dL [7], and the diagnostic guidelines use a value higher than 1.1 times the upper reference limit as a diagnostic indicator. In addition, as mentioned above, serum IgG levels are often low in acute and severe cases, so caution should be exercised [7]. The clinical characteristics of AIH cases in East Asia are changing, with serum IgG levels and rates of anti-nuclear antibody positivity decreasing [18]. Acute-onset AIH often lacks the findings characteristic of AIH, and some cases were misdiagnosed as DILI and progressed to liver failure [8]. Interface hepatitis with mononuclear cell infiltration is a frequently observed histology in AIH and is often accompanied by plasma cell infiltration [19]. Hepatocellular necrosis within the lobules, hepatocellular rosette formation, and emperipolesis are also observed in many cases [19]. When other causes of liver injury are excluded, hepatocellular rosette formation and emperipolesis have been reported to have diagnostic utility for AIH [19].

#### 2.3.2. AIH and International Diagnostic Criteria

The revised international diagnostic criteria for AIH [20] have excellent diagnostic sensitivity and can pick up and diagnose even atypical cases in which findings such as autoantibody positivity and high IgG levels are not prominent. On the other hand, the simplified international diagnostic criteria [21] have been validated by clinical practice studies, as previously demonstrated [22,23], have excellent diagnostic specificity, and are useful in differentiating AIH-like cases from true AIH cases [22,23]. Therefore, it is important to keep in mind that the simplified international diagnostic criteria may miss atypical cases, including acute-onset cases, and to use both diagnostic criteria as appropriate for each case.

#### 2.3.3. Acute AIH and IgG4-Related AIH

Currently, acute hepatitis-like AIH is divided into two pathological phases: acute exacerbation-phase AIH and acute hepatitis-phase AIH [24]. Acute exacerbation is a well-known clinicopathological concept in AIH and chronic hepatitis B [24]. It is an acute hepatitis-like exacerbation during the course of chronic hepatitis. Acute hepatitis-phase AIH is an acute onset case with no preceding chronic hepatitis (or subclinical) [24]. In recent years, the recognition of such acute hepatitis-phase AIH has become widespread, and the number of cases of acute hepatitis requiring differentiation from DILI has increased rapidly, leading to more cases of acute hepatitis being subjected to liver biopsy [8]. Acute-onset AIH is characterized by an acute hepatitis picture showing diffuse hepatocellular damage, especially in cases with inflammation around central veins leading to lobular central zonular necrosis (centrilobular necrosis), a regional necrosis [8]. There are also many acute onset AIH cases with plasma cell infiltration, hepatocellular rosette formation, and emperipolesis, which also appear in idiopathic AIH [8]. In about 3% of patients with AIH, there are cases with elevated serum IgG4 levels and histological evidence of numerous IgG4-positive plasma cell infiltrates in the portal area. Some cases are complicated by IgG4-related sclerosing cholangitis and type 1 autoimmune pancreatitis, which are referred to as IgG4-related AIH, a liver-specific IgG4-related disease [25]. As for treatment in IgG4-related AIH, immunosuppressive therapy with corticosteroids is the first choice, as in AIH and other IgG4-related diseases. IgG4-related AIH has been reported infrequently, and the differences between typical AIH and acute AIH are an issue for further study. Acute patients with AIH may develop acute-on-chronic liver failure due to infection, excessive alcohol intake, and drugs [26]. Thus, prudent use of any medication should be stressed in the clinical management of patients with AIH.

### 2.4. Treatment of AIH

#### 2.4.1. Treatment for AIH and Prognosis

The AASLD guidelines define response to treatment as: (1) biochemical remission—normalization of serum alanine aminotransferase (ALT), aspartate aminotransferase, and IgG levels; (2) histological remission—resolution of inflammation in liver tissue after treatment; (3) incomplete response—improvement of laboratory findings that do not lead to biochemical remission; (4) treatment failure—worsening of laboratory and tissue findings during treatment; (5) treatment intolerance—inability to continue treatment due to drug-related side effects; and (6) relapse—worsening of disease activity after biochemical remission [27]. In Japan, serum transaminases improve in more than 90% of patients with AIH treated with corticosteroids. Additional administration of azathioprine (1–2 mg/kg/day) to patients whose serum transaminases are not controlled within the reference range by corticosteroid therapy or who relapse during treatment results in remission in more than 90% of cases [28]. In Japan, a public knowledge application for azathioprine in AIH was approved in July 2018 [28]. In cases with good response to treatment with corticosteroids at the time of initial therapy, increasing or resuming corticosteroids is also effective at the time of relapse [27]. Even if anti-mitochondrial antibodies are positive, corticosteroid therapy should be considered if the score by the simplified international diagnostic criteria [21] is above the threshold for suspicion of AIH. The rate of anti-mitochondrial antibody positivity in AIH is about 10% [27]. The long-term prognosis of AIH cases in Japan is reported to be good, with a 10-year survival rate of more than 95%, which is not different from that of the general population [28]. It is important to note that both corticosteroids and azathioprine have a wide variety of side effects. Azathioprine-related side effects occur in approximately 10% of patients with AIH [29]. The development of second-line treatment for patients with AIH who are intolerant or refractory to corticosteroids and azathioprine is awaited. On the other hand, moderate depression is found in 19% and severe depression in 10% of patients with AIH and is strongly associated with fatigue. Patient anxiety may affect the treatment of AIH [30]. Increased intestinal permeability has been reported in patients with AIH, and the expression of tight junction-related proteins, ZO-1 and occludin, decreases as AIH becomes more severe [31]. In animal studies, probiotics for AIH have been reported to improve AIH via improved intestinal permeability [32].

#### 2.4.2. AIH, Azathioprine, and NUDT 15 Gene Polymorphism

Among the side effects of azathioprine, severe acute leukopenia and total hair loss that occur early after initiation of azathioprine have been associated with the Nudix hydrolase (NUDT) 15 gene polymorphism related to the metabolism of azathioprine [29]. However, it should be noted that even in cases of genotypes that are considered relatively safe, side effects such as decreased blood cell counts are not uncommon [29]. The frequency of the NUDT15 Arg139Cys gene polymorphism is reported to be about 1% in homozygotes (Cys/Cys) and about 20%in heterozygotes (Arg/Cys, Cys/His). In a survey on NUDT15 Arg139Cys polymorphisms and adverse events in 1291 Japanese patients with inflammatory bowel disease previously treated with thiopurines, leukopenia was reported in 45 of 49 patients and hair loss in 46 of 49 patients with the Cys/Cys genotype, and leukopenia was reported in 94 of 275 patients with the Arg/Cys genotype; alerts have been issued [33]. Therefore, when newly using azathioprine, the use of azathioprine should, in principle, be avoided in the case of the Cys/Cys type in the NUDT15 genotype test (in Japan, insurance coverage has been available for AIH since November 2019) because the risk of serious side effects is very high. On the other hand, in the case of Arg/Cys and Cys/His, the use of azathioprine should be considered starting at low doses. It should be noted that even in the case of Arg/Arg and Arg/His types, which are considered to have a low risk of leukopenia and hair loss, regular monitoring of adverse effects is necessary [33].

#### 2.4.3. AIH and Pregnancy and UDCA Therapy

AIH is linked to a significant increase in maternal pre-pregnancy and gestational diabetes, and women with AIH are more likely to have premature births, small for gestational age births, and babies with lower birth weight, and they show a significant decrease in full-term birth compared to controls [34]. On the other hand, no difference in miscarriage or birth rates has been reported between patients with AIH treated with corticosteroids or azathioprine at the time of pregnancy diagnosis and those who were untreated [35]. In many cases, the disease state of AIH stabilizes during pregnancy. However, AIH flares up or worsens in 10–20% of cases during pregnancy and in 10–50% of cases after delivery, especially within 3 months after delivery [35,36]. Although azathioprine is no longer contraindicated for pregnant women in 2018 in Japan, azathioprine should only be administered during pregnancy if the therapeutic benefit is judged to outweigh the risks, and pregnancy should be avoided while taking this drug whenever possible. Ursodeoxycholic acid (UDCA) should also not be administered. For UDCA in AIH, UDCA 600 mg/day is generally used [37]. If transaminases are not controlled within the reference range with UDCA alone, corticosteroid administration should be considered [37]. In addition, concomitant use of UDCA during corticosteroid administration may assist in corticosteroid dose reduction, but clinical evidence has not been established. Although UDCA is frequently used for patients with AIH in Japan, it is not mentioned as a treatment option for AIH in AASLD practice guidelines [38] or EASL guidelines [39]. Compared to corticosteroid therapy, UDCA monotherapy requires a longer period of time from the start of treatment for improvement of serum transaminases to within the reference range, so UDCA monotherapy is not recommended for patients who require rapid improvement of serum transaminases, such as patients with cirrhosis.

#### 2.4.4. When to End Treatment for AIH

Termination of corticosteroid therapy for patients with AIH can be considered in cases in which serum transaminases and IgG have been maintained within the reference range continuously for more than 2 years with corticosteroid therapy. However, since relapse occurs within 3 years in most cases in which corticosteroid treatment is terminated, adequate follow-up is necessary even after treatment is completed [40]. On the other hand, it should be noted that even in patients with serum transaminases within the reference range, inflammatory findings in liver tissue have been reported in 55% of cases during treatment and in 20% after the discontinuation of treatment [41].

#### 2.4.5. AIH and Liver Transplantation

Liver transplantation (LT) is an effective treatment when medical therapy fails to provide sufficient benefit and leads to decompensated cirrhosis or when the disease develops as acute liver failure. Decompensated cirrhosis allows patients with a Child–Pugh score of 10 or higher to be placed on the Organ Transplant Network’s brain death LT waiting list. LT is also effective when acute onset cases become fulminant or lead to late-onset liver failure. The results of LT for AIH in Japan are favorable, with a 10-year survival rate of 75%, which is comparable to the results of LT for other diseases [42]. There is no apparent difference in LT-related outcomes between deceased donors and living donors. AIH may recur after LT, but medical therapy is effective, as is usual for AIH. AIH recurrence of 8–12% at 1 year post-LT and 36–68% at 5 years post-LT have been reported [43]. Risk factors for AIH relapse after LT have been reported, including young age, post-LT use of mycophenolate mofetil, donor and recipient sex mismatch, and higher pre-LT IgG levels [44].

### 2.5. COVID-19 Vaccine and AIH

It is speculated that SARS-CoV-2 may disrupt self-tolerance and cause autoimmune reactions, and in fact, there are increasing reports of autoimmune diseases that develop after infection with SARS-CoV-2, such as Guillain–Barre syndrome and primary biliary cholangitis [45]. It has been suggested that the COVID-19 vaccine may cause similar reactions [46,47], but such cases are extremely rare in Japan. As viral spike proteins have been implicated in the immune system, vaccination-induced spike proteins may cause autoimmune diseases [48]. In general, DI-ALH, such as that caused by minocycline, often shows a long incubation period of 6 months to more than a year, whereas most cases of AIH caused by the COVID-19 vaccine are seen as early as 1 week or less [49].

## 3. DILI

### 3.1. Clinical Features and Diagnosis for DILI

DILIs are classified into intrinsic DILIs, which are dose-dependent and predictable, and idiosyncratic DILIs, which are dose-independent and unpredictable [50,51]. Intrinsic DILI is a liver injury caused by the metabolite of a drug taken in excess of a certain dose, regardless of physical constitution, and acetaminophen is a typical causative agent [50]. On the other hand, in idiosyncratic DILI, drug metabolites acquire immunogenicity and induce cytotoxicity by immune cells. Many DILIs are caused by the idiosyncratic DILI, and HLA polymorphisms are involved in their pathogenesis [51]. The chemical properties of drugs are important in triggering DILI, and fat-soluble drugs that easily pass hepatocyte membranes are at higher risk of developing DILI. Drugs that undergo hepatic metabolism are also susceptible to DILI. As for intrinsic DILI, reactive intermediate metabolites metabolized by cytochrome P450 in the liver directly cause hepatocyte necrosis [50]. As for idiosyncratic DILI, in humans with a genetic predisposition such as HLA polymorphisms, reactive intermediate metabolites activate the acquired immune system and cause hepatotoxicity by cytotoxic T cells [51]. Idiosyncratic DILI with skin rash and elevated eosinophils is rather exceptional because it is not due to an allergic reaction involving IgE release [51]. However, although rare (about 500 cases per year in Japan), drug-induced hypersensitivity syndrome (DIHS) with eosinophilia and severe systemic symptoms should be noted [52].

The following factors are important in the diagnosis of DILI: (1) a detailed history of drug intake, (2) an accurate understanding of the temporal relationship between the start or end date of drug intake and the course of liver injury, and (3) exclusion of liver injury due to other causes [53]. Re-administration of the suspected drug for definitive diagnosis is contraindicated. It should be recognized, however, that this is dependent on the indications for the drug and available alternatives. Regarding the temporal relationship between drug administration and liver injury, the general rule is that liver injury appears after drug administration and recovers upon discontinuation, but it may take various time courses depending on the drug and the type of liver injury and may develop after discontinuation of the suspected drug [53,54]. In addition, DILI may occur in patients who already have liver damage due to other causes, such as fatty liver, alcoholic liver disease, chronic viral hepatitis, or AIH, and these cases should be treated with caution. DILI is not always easy to diagnose because (1) the clinical presentation of DILI is diverse, ranging from minor liver injury to acute liver failure; (2) there are few sensitive and specific biomarkers or pathological findings for DILI; and (3) in many cases, a variety of drugs are used in combination [55]. The extremely low incidence of DILI with certain drugs, except for those drugs that cause DILI frequently, also makes it difficult to collect cases and elucidate the pathogenesis of DILI.

### 3.2. Assessment Method for DILI

The site most susceptible to DILI is zone 3 (the central lobular region), which is rich in drug-metabolizing enzymes [56]. DILI is classified into hepatocellular damage type, biliary stasis type, and mixed type, depending on the degree to which ALT and alkaline phosphatase (ALP) exceed the upper limit of the reference values [53,54]. R is defined as the value obtained by dividing the “ratio of ALT value to the upper reference limit” by the “ratio of ALP value to the upper reference limit,” and R is defined as hepatocellular damage type if R is 5 or higher, biliary stasis type if R is 2 or lower, or mixed type if R is between 2 and 5 [54].

**Figure 1:**
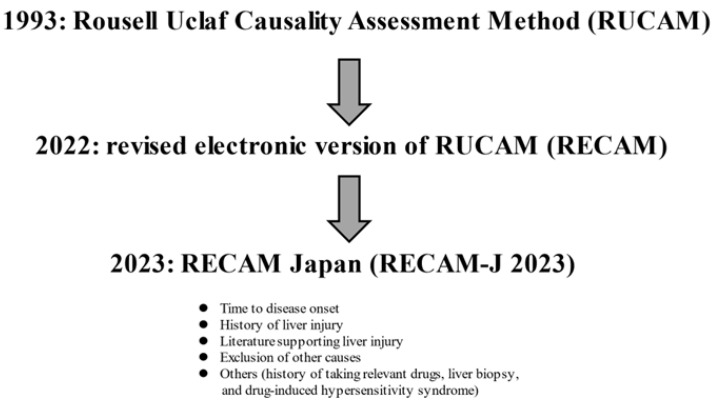
Evolution of diagnostic criteria for DILI.

A scoring system called the Rousell Uclaf Causality Assessment Method (RUCAM) was developed in 1993 [57]. In 2022, a new scoring system, the Revised Electronic Version of RUCAM for the Diagnosis of DILI (RECAM), based on the US and Spanish DILI registries, was reported [53]. A Japanese version (RECAM-J 2023) has also been proposed [58]. The RECAM-J 2023 consists of five categories: (a) time to disease onset, (b) history of liver injury, (c) literature supporting liver injury, (d) exclusion of other causes, and (e) others (history of taking relevant drugs, liver biopsy, and DIHS) (Figure 1). The possibility of DILI diagnosis is evaluated by summing the scores of each category. In a study of the usefulness of RECAM-J 2023 in Japanese patients, the area under the characteristic curve (AUC) value for the “very likely” and “likely” diagnoses was 0.88 [58]. Intrinsic DILI due to acetaminophen, etc., and liver injury due to ICIs are not covered by RECAM-J 2023. On the other hand, it is particularly important to distinguish AIH in the hepatocellular damage type of DILI and acute cholangitis in the cholestasis type of DILI. In cases in which liver biopsies were performed, zonular necrosis and acute and chronic inflammation were reported to be the main findings of liver histology in the hepatocellular damage type of DILI, and acute and chronic biliary stasis in the biliary stasis type of DILI [59]. Estrogen and tamoxifen are also known to cause steatohepatitis-like pathology [60]. Anabolic hormones and oral contraceptives are known to cause focal nodular hyperplasia [61] and hepatocellular adenomas [62] (mass-forming DILI).

### 3.3. ICIs and Liver Injury

A review of 307 DILI cases collected from 2010 to 2018 in Japan reported that anti-inflammatory drugs and antibiotics were the most frequent, followed by anticancer agents and other drugs [63]. DILI is expected to increase with the remarkable development of new drugs in recent years. Liver injury due to ICIs is a special form of DILI [64]. Based on the liver histology and the course of the disease after discontinuation of ICIs, it is clearly different from AIH [65]. ICIs activate T cells, especially CD8-positive T cells, which, together with macrophages infiltrating the liver, induce liver injury [64]. The incidence is high when multiple ICIs are administered in combination, especially when anti-CTLA-4 antibodies are used in combination, which is often severe. The timing of the onset of hepatotoxicity depends on the drug. Liver injury occurs 6–14 weeks after the initiation of treatment with anti-PD-1/PD-L1 antibody monotherapy, but it often appears even earlier with anti-CTLA-4 antibody combination therapy [66,67]. The basic pathophysiology of liver injury due to ICIs is acute hepatitis-like hepatocellular damage and necro-inflammatory changes with lymphocytic infiltration in the lobules [64]. Eosinophils are also prominent in about 20% of cases, so it is important to distinguish this from the common DILI. In addition to Kupffer cell swelling, granulomatous inflammation due to the infiltration of histiocytes is prominent in the liver sinusoids [68]. If liver injury occurs during ICI administration, other causes of liver injury (exacerbation of intrahepatic neoplastic lesions, common DILI, fatty liver, etc.) should be ruled out first. Then, depending on the severity of the disease, therapeutic measures such as continued ICI administration, ICI withdrawal, corticosteroid administration, and additional administration of mycophenolate mofetil are selected with careful follow-up [65]. Another condition that can cause liver injury during ICI treatment is cholangitis with non-obstructive bile duct dilation and thickening of the bile duct wall [69]. Most cholangitis due to ICI is classified as non-hepatocellular damage, and a poor response to corticosteroids has been reported [70]. For liver injury caused by ICIs, it is essential for gastroenterologists and physicians of various departments to collaborate in early diagnosis and appropriate treatment.

### 3.4. Treatment for DILI

As for the treatment for DILI, the main principle is to discontinue the suspected drug, and in many cases, the disease resolves with the discontinuation of the drug alone [71]. Since the causal relationship between the drug under investigation and liver injury may be established by the fact that the patient has recovered after discontinuation of the drug alone, it is preferable to follow the patient without treatment if possible but to pay close attention to signs of severe disease, such as jaundice and prolonged prothrombin time [71]. If multiple drugs are being administered and the liver injury is relatively mild, the drugs should be discontinued in order of likelihood, referring to past adverse drug reaction reports to determine the causative drug. If liver injury is moderate to severe, all drugs that can be discontinued should be discontinued at the same time. If there is no improvement in liver injury after discontinuation of the drug, a careful search for other causes of liver injury, such as AIH, should be conducted again. Although hepatoprotective drugs are sometimes administered for DILI in Japan, there is no evidence of efficacy, and hepatoprotective drugs should not be administered without careful evaluation of other possible causes [71]. N-acetylcysteine is effective in acetaminophen-induced DILI [50].

## 4. DI-ALH

DILI can have clinical features similar to AIH (DI-ALH) and should always be considered as a differential disease [4]. DI-ALH should be distinguished from idiopathic AIH, which requires continuous immunosuppressive treatment, but there is currently no established biomarker specific for DI-ALH [4]. The definition of DI-ALH in the EASL Clinical Practice Guideline is “acute DILI with serological and/or histological markers of idiopathic AIH” [4,71]. The pathogenesis of DI-ALH includes the following: (1) liver-specific autoantigens, such as liver-specific protein and liver membrane antigen released from hepatocytes upon DILI, may induce AIH; (2) specific autoantibodies are produced against cytochrome P450, which is expressed on hepatocyte membranes, and may cause hepatocellular damage; (3) drug metabolites become haptens and bind tightly to cellular components to acquire antigenic properties, which may induce hepatocellular damage through cellular immune mechanisms against these hapten carriers [72,73]. Causative drugs known to induce DI-ALH include minocycline, infliximab, statins, interferon, fenofibrate, etc. [10]; however, the exact frequency for each drug is unknown.

Although the low risk of relapse after long-term discontinuation of immunosuppressive treatment is a characteristic of DI-ALH [74], the timing of initiation, duration, and discontinuation of immunosuppressive treatment in DI-ALH is largely empirical, and there is no clear evidence for these. At the very least, one should keep in mind the drugs that can cause DI-ALH [4,74]. In DI-ALH, if liver enzymes do not spontaneously recover, the patient should be treated with corticosteroids rather than azathioprine in the first round, and only if the patient still relapses (rare in DI-ALH) should azathioprine be considered for addition [74].

## 5. Final Remarks

**Figure 2:**
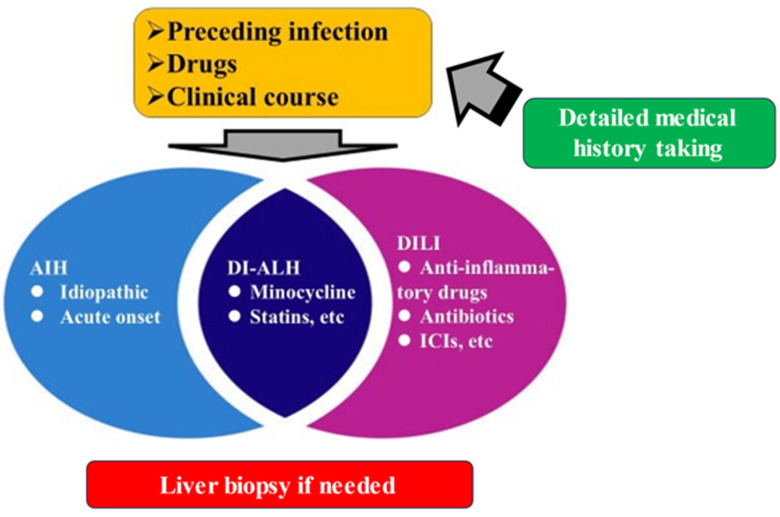
The relationship between AIH, DI-ALH, and DILI.

AIH and DILI in Japan were outlined in this article. Situations vary, including cases that are easily diagnosed and cases in which it is difficult to differentiate between the two. In recent years, acute-onset AIH and liver damage due to ICIs have been increasing in Japan. It is important to understand the differences and pathological features between these two types of liver injury and idiopathic AIH in order to make a differential diagnosis. Finally, the authors’ proposed relationship between AIH, DI-ALH, and DILI is shown in Figure 2.

## Abbreviations

AIH; autoimmune hepatitis, DILI; drug-induced liver injury, ICI; immune checkpoint inhibitor, DI-ALH; drug-induced AIH like hepatitis, LKM; liver kidney microsome, SLA; soluble liver antigen, ALT; alanine aminotransferase, NUDT; Nudix hydrolase, UDCA; ursodeoxycholic acid, LT; liver transplantation, DIHS; drug-induced hypersensitivity syndrome, ALP; alkaline phosphatase, RUCAM; Rousell Uclaf Causality Assessment Method, RECAM; Revised Electronic Version of RUCAM for the Diagnosis of DILI, ICI; immune checkpoint inhibitor
